# Moving towards in pouch diagnostics for ostomy patients: exploiting the versatility of laser induced graphene sensors

**DOI:** 10.1007/s10853-023-08881-x

**Published:** 2023-09-08

**Authors:** Conor McCann, Victoria Gilpin, Cameron Scott, L. Kirsty Pourshahidi, Chris. I. R. Gill, James Davis

**Affiliations:** 1 School of Engineering, Ulster University, Belfast, Northern Ireland; 2 School of Biomedical Sciences, Ulster University, Coleraine, Northern Ireland

## Abstract

The development of a 3D printed sensor for direct incorporation within stoma pouches is described. Laser induced graphene scribed on either side of polyimide film served as the basis of a 2 electrode configuration that could be integrated within a disposable pouch sensor for the periodic monitoring of ileostomy fluid pH. The graphene sensors were characterised using electron microscopy, Raman spectroscopy, DekTak profilometry with the electrochemical properties investigated using both cyclic and square wave voltammetry. Adsorbed riboflavin was employed as a biocompatible redox probe for the voltammetric measurement of pH. The variation in peak position with pH was found to be linear over pH 3–8 with a sub Nernstian response (43 mV/pH). The adsorbed probe was found to be reversible and exhibited minimal leaching through repeated scanning. The performance of the system was assessed in a heterogeneous bacterial fermentation mixture simulating ileostomy fluid with the pH recorded before and after 96 h incubation. The peak profile in the bacterial medium provided an unambiguous signal free from interference with the calculated pH before and after incubation (pH 5.3 to 3.66) in good agreement with that obtained with commercial pH probes.

**Graphical abstract:**

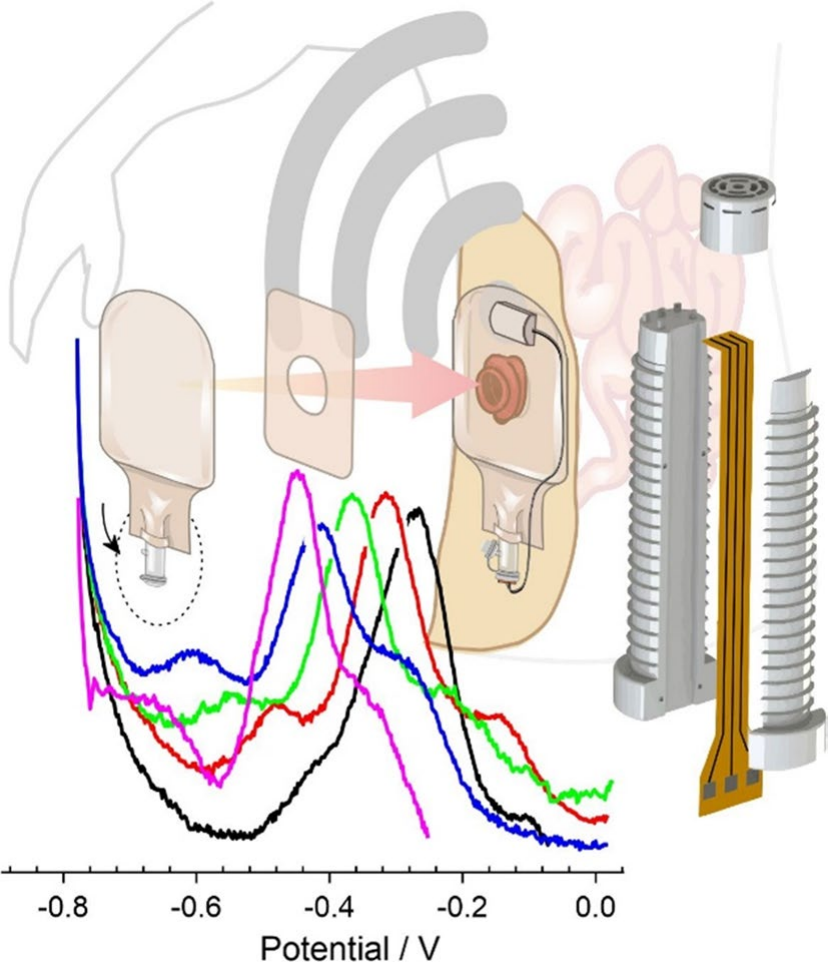

**Supplementary Information:**

The online version contains supplementary material available at 10.1007/s10853-023-08881-x.

## Introduction

Colorectal conditions such as inflammatory bowel disease, trauma, diverticular disease, and malignancy can require surgical intervention and intestinal diversion through the creation of a stoma. The latter is a surgical opening on the abdomen through which the passage of faeces is directed into an external disposable pouch [1]. It has been estimated that there are between 175000 and 205000 ostomates (ileostomy, colostomy and urostomy) in the UK, with over 20000 new stomas created each year [2, 3]. While there have been substantial advances in surgical procedures, complications post release remain considerable and can impact on psychological and physiological wellbeing. The creation of a diverting ileostomy can be particularly problematic where the intestinal surface area for reabsorption can be greatly reduced thereby leading to a high stomal output [4, 5]. Where there is persistent loss of large amounts of small bowel fluid (1500–2000 mL/24 h), dehydration and electrolyte disorders can arise and are a common factor in the readmission of patients with an ileostomy [6–9]. High output stomas (HOS) can also greatly increase the propensity towards pouch leakage [10, 11] and peristomal skin complications [12]. Moisture associated skin damage through prolonged exposure to the bowel fluid can be particularly troublesome for patients as it can directly impact on the subsequent adhesion of the collection pouch to the skin [13]. It is perhaps little surprise that the composition of the faecal fluid could be a rich source of diagnostic information, providing insight to the gastrointestinal, renal and dermatological wellbeing of the patient. The ability to monitor key biomarkers such as pH, electrolytes or inflammatory markers (i.e. calprotectin) at the point of care rather than at scheduled consultations could offer greater opportunities to optimise the care pathway for those with HOS [14, 15].

**Figure 1:**
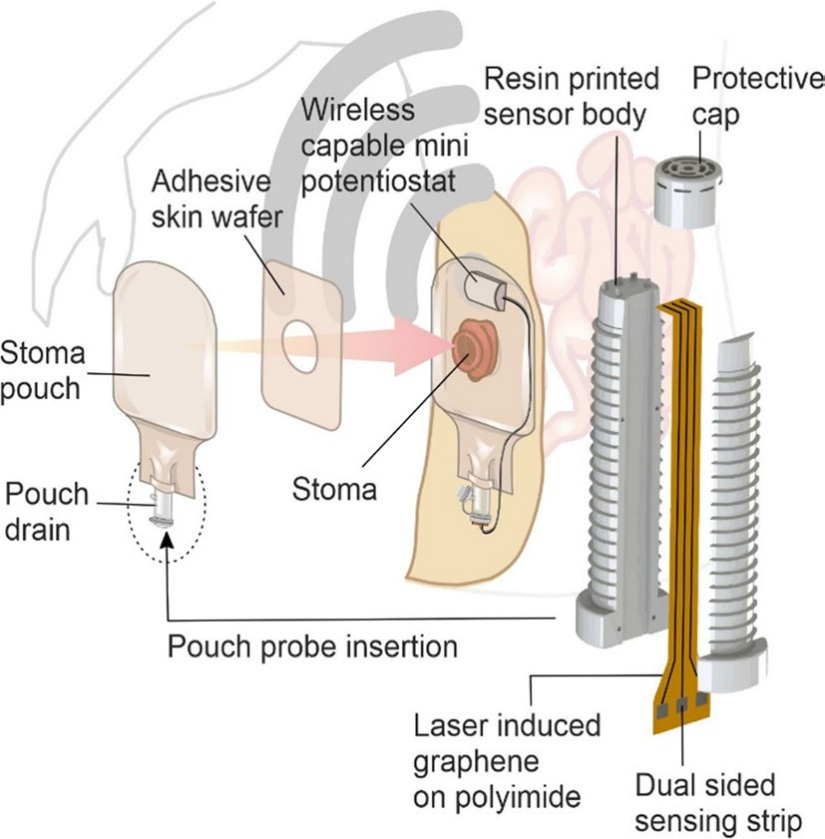
Design methodology for in pouch autonomous monitoring.

As sensing moves towards precision medicine, it could be envisaged that ileostomy fluid data could be used to target more effective dietary management and therapeutic prescription. There are however substantial challenges to pursuing such a strategy where the competence of the patient (or carer) and capability of the technology to perform the required measurements are questionable. While it could be envisaged that a sensing strategy similar to home glucose monitoring (HGM) could be adopted, adherence to a regular sampling regime could be problematic. Such issues are common in HGM [16, 17] and could be greatly exacerbated in this scenario when considering the nature of the proposed sample. Autonomous sensing that required no user interaction beyond the simple insertion of the sensing component within the pouch space would be a much more ideal concept. This gives rise to a number of key issues: the implementation of the sensor within the pouch and the subsequent acquisition of a robust and accurate signal within a highly complex, variable and heterogeneous fluid. The sensing strategy is further complicated by the fact that the typical wear time for a pouch is around 5 days [18] (though it could be less than a day for those patients experiencing frequent leakage) and, as such, would need to be based around a disposable sensing strip. The aim of the present communication has been to explore a potential solution to these challenges through the use of a composite design incorporating a 3D printed insert within which laser induced graphene tracks serve as the sensing substrate. The core design rationale is highlighted in Fig. 1 where the insert would be placed within the drainage tap of a conventional/commercial stoma pouch. In doing so, the probe would have direct access to the internal pouch fluid without having to compromise the normal function of the bag itself.

Laser induced graphene (LIG) has no direct selectivity towards a given biomarker but it provides a rapid prototyping option that is scalable with the conductive substrate allowing additive tailoring to a specific application. In this communication, the measurement of pH was selected as a suitable model system through which to evaluate the efficacy of the design and performance of the underpinning LIG sensor. Modification of the carbon surfaces with flavin derivatives through either electropolymerisation [19, 20] or simple physisorption [21] has been shown to provide a versatile option for voltammetric pH sensing and it was envisaged that a similar strategy could be adopted here. The production and characterisation of LIG-based riboflavin sensors integrated within the 3D printed insert is reported and the ability to operate within a complex heterogeneous bacterial mixture is critically assessed.

## Experimental details

All reagents were of the highest grade available and obtained from Sigma and were used without further purification. Polyimide film used in the fabrication of the electrodes was 125 µm thick, Dupont Kapton^®^ (4-100-KHN-5, TapeCase, IL, USA). Britton–Robinson (BR) buffer solutions were used throughout and were composed of equimolar acetic, phosphoric and boric acids (0.04 M) adjusted to the required pH through the addition of NaOH. The buffer solutions were supplemented with 0.1 M KCl in order to define the potential of the Ag|AgCl pseudo reference employed in the probe investigations. Electrochemical analyses were conducted using a PalmSens Emstat 3, wireless enabled portable potentiostat with initial investigations employing a 3-electrode configuration where laser induced graphene served as the working electrode, platinum wire as the counter electrode and a commercial Ag|AgCl half cell (3 M NaCl) reference. Later studies involving the 3D printed pouch probe (Fig. 1) employed a 2-electrode configuration with laser induced graphene serving as the base substrate for both. Voltammetric analysis was conducted at 22 °C ± 2 °C without solution degassing.

### Preparation and characterisation of LIG probe

**Figure 2:**
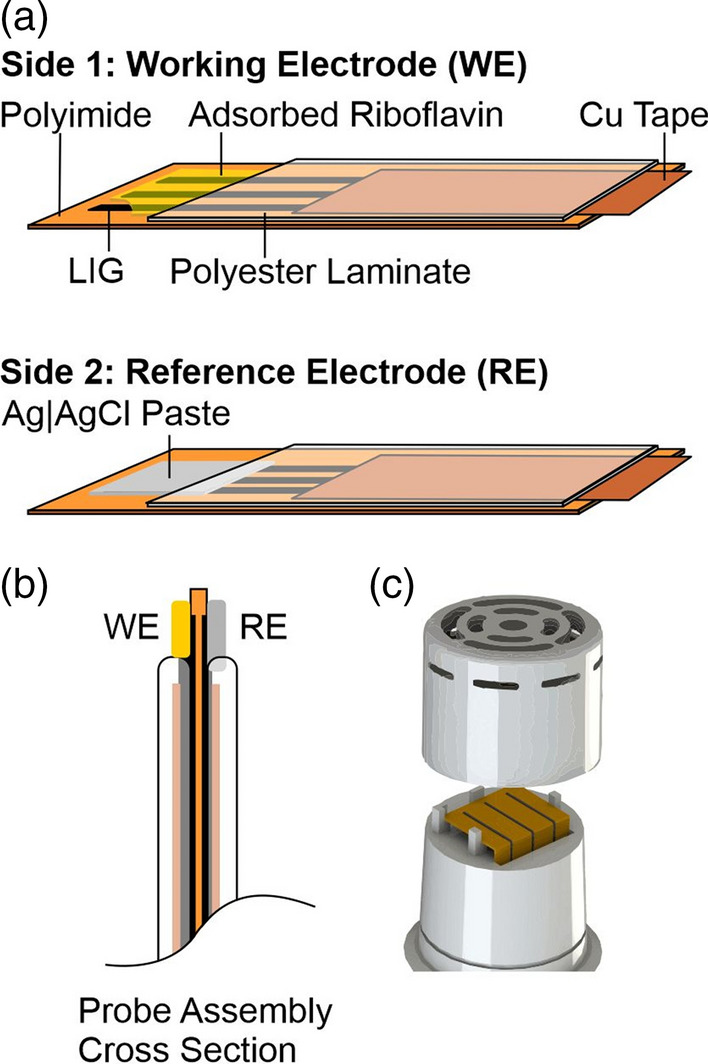
**a** Individual components of the working (WE) and reference (RE) electrodes. **b** Cross section highlighting the dual layer nature of the probe. **c** The positioning of the sensing head within the probe body and protective cap.

The LIG substrates were produced by directly scribing the polyimide in an air atmosphere using an Atomstack A5 Laser, 5W Laser Diode, (445 nm) operating at 20% with a raster speed of 4000 mm/min. The line density was 20 lines/mm and each design was subject to two passes. The LIG samples were initially sectioned into 5 mm × 5 mm squares and an adhesive copper tape added to the bottom to serve as the external contact. The LIG-Cu track was subsequently thermally encapsulated into polyester laminates that had been precut with a 3 mm × 3 mm square window to expose the LIG surface and control the geometric area of the working electrode in a manner similar to that reported by Casimero et al. [19, 22]. The 2-electrode probe used with the 3D printed sensor body (Fig. 1) employed LIG tracks scribed on either side of a single sheet of polyimide—each side mirroring the other side. The working electrode consisted of three discrete LIG tracks (each 500 μm wide × 6.7 cm long) but co-connected via a single copper track as indicated in Fig. 2a. The polyimide was then laminated with only the end 5 mm of each LIG track exposed providing a combined working area of 7.5 mm^2^. Riboflavin was physi-sorbed onto the LIG through drop casting (50 μL) from a saturated riboflavin/methanol solution and allowed to dry before being rinsed with fresh methanol. Silver–silver chloride paste was applied to the exposed LIG on the opposite side of the polyimide strip and laminated as before and served as the combined counter/pseudo reference. The dual sided nature of the probe design is highlighted in Fig. 2b. The polyimide was then glued into the 3D printed probe body and the sensing head enclosed with a protective cap as indicated in Fig. 2c. The design specifications of the probe body and cap are detailed in the supporting information (Figures S1 and S2) along with electron micrographs of the pillars that support the positioning of the LIG sensing strip (Figure S3).

The drain inserts were printed using a Creality 4 K LD-006 (UV-LCD) resin printer using an AnyCubic White resin (405 nm). Raman spectra of the LIG samples were obtained using a Renishaw Raman Microscope (20 × objective lens) with a 532 nm laser operating at 10% power. Conductivity measurements of the lasered polyimide were acquired with an Ossila Four Point Probe.

## Results and discussion

### LIG characterisation

**Figure 3:**
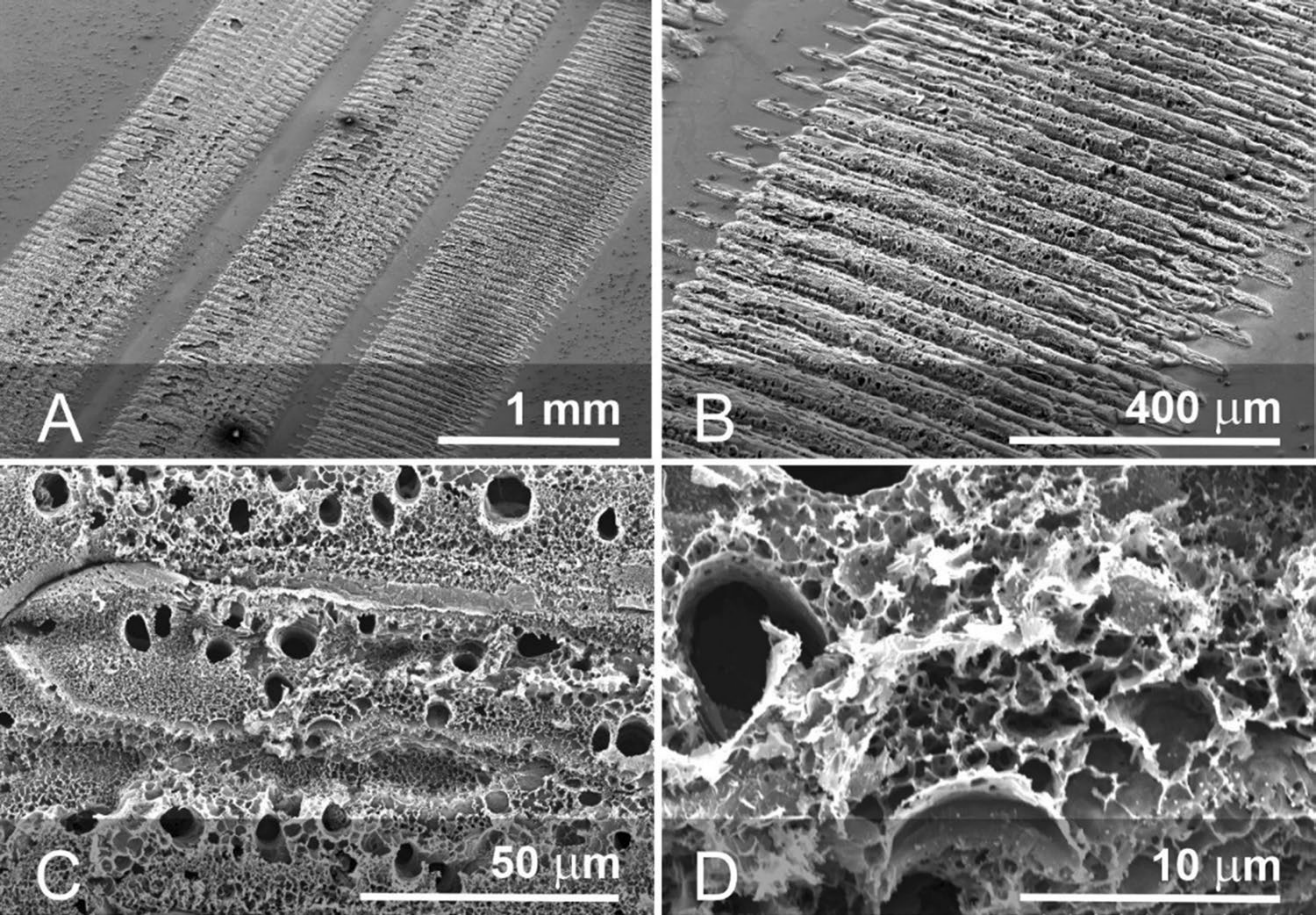
Electron micrographs detailing the LIG morphology arising from the x-y raster process (**a**, **b**) closer examination of the micro-nano porous carbon deposit (**c**, **d**).

The surface morphology of the LIG electrodes created from polyimide sheet was investigated using scanning electron microscopy and representative images are highlighted in Fig. 3. While the PI surface is smooth and featureless, laser scribing results in a series of raised carbonised furrows which appear to be orientated perpendicular to the direction of the track creating a ribbed morphology (Figs. 3a and b). The latter is a characteristic artefact of the diode laser function where the LIG is formed from discrete pulses rastered across the pre-programmed pattern rather than the continuous sweep methodology employed with CO_2_ lasers. More detailed examination of the carbon deposit reveals a foam like architecture and can be attributed to the intensity of the localised heat leading to the degradation of the underlying polymer with the consequent liberation of gas raising the carbonised material. While the surface can be seen to possess a heterogeneous morphology with a spectrum of micro-nano porosity (Figs. 3c and d) which is broadly consistent with the LIG morphology observed by others [23, 24].

**Figure 4:**
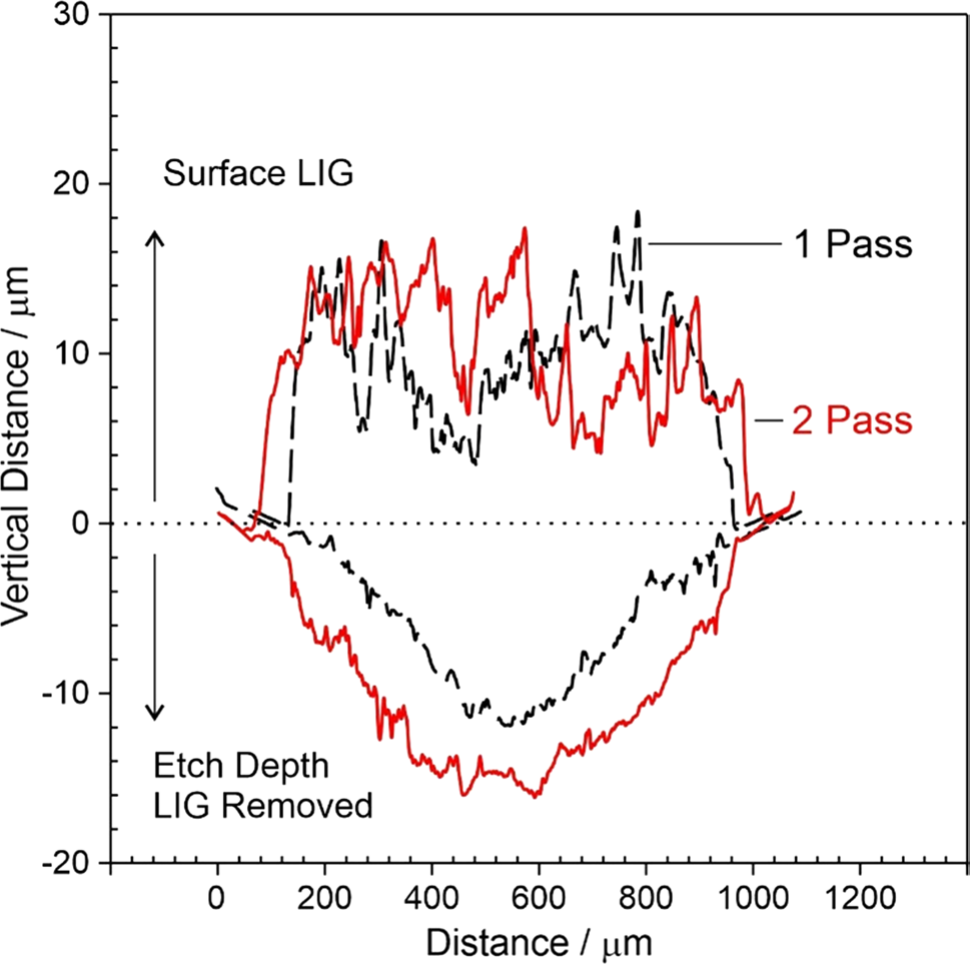
Surface profiling across laser tracks formed from either a single or double pass. Subsequent removal of the LIG deposit via sonication allowed the etch depth to compared.

It can be seen from the electron micrographs detailed in Fig. 3 that the surface of the carbonised track is highly heterogeneous with crater like regions dispersed throughout where the sections of the polymer have been ablated. The effect of multiple passes on the extent of LIG formation on the PI was investigated by examining cross sections of the lasered tracks using DekTak profilometry. The height and depth of the LIG layer arising from single and double laser passes are compared in Fig. 4 and the summary statistics presented in Table 1. While there is considerable variation in the height along each track, the second pass does not substantially influence the mean height of the LIG layer. In contrast, both the etch depth and breadth are notably increased by employing the second pass. Given that the thickness of the polyimide is 125 μm, the use of double pass ablation allowed the proposed dual layer probe designs (Sect. "Preparation and characterisation of LIG Probe"**, **Fig. 2) to be pursued without issues of the laser co-connecting LIG layers on either side.

**Table 1:** Surface profile characteristics arising from single or dual pass laser ablation

	LIG height/µm	Etch depth/µm	*N*
1 Pass	20.2 ± 1.2	14.1 ± 0.52	9
2 Pass	17.6 ± 0.97	19 ± 0.92	9

**Figure 5:**
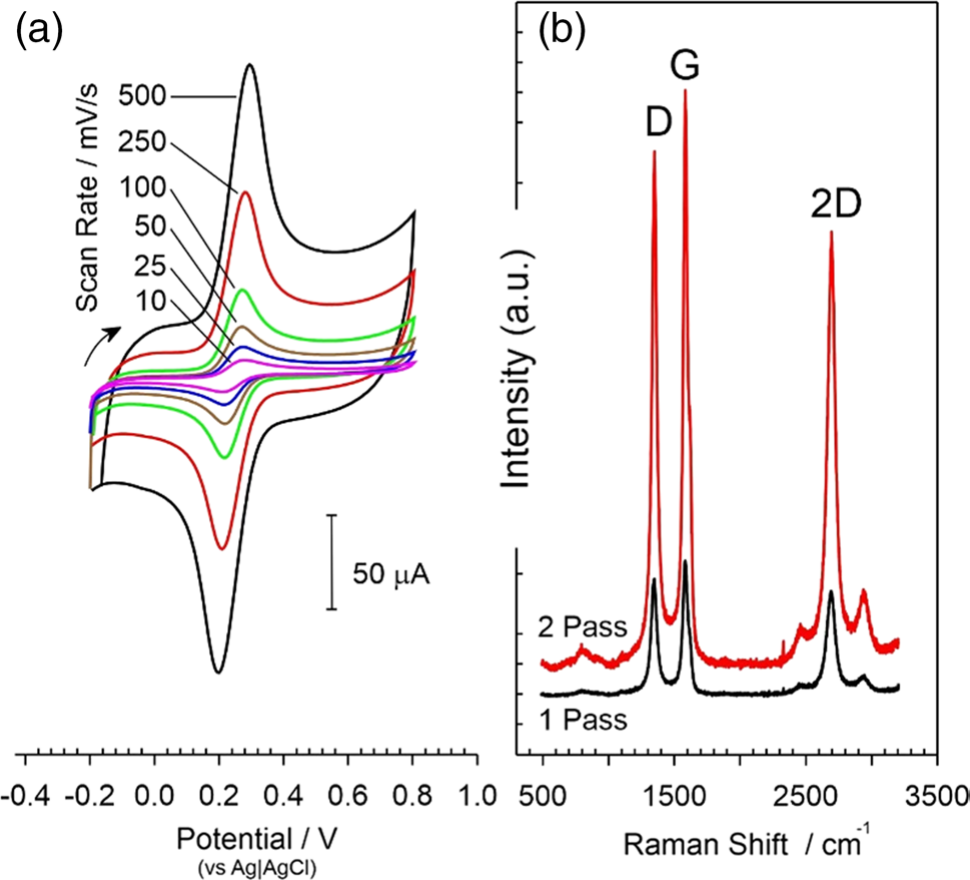
**a** Cyclic voltammogram detailing the response of a 2-pass LIG electrode to ferrocyanide (2 mM, pH 7, 50 mV/s) and **b** Raman spectrum of the LIG substrate after single and double pass ablation scans.

The electrochemical properties of the LIG electrodes were assessed using a ferrocyanide redox probe (2 mM, pH 7, 50 mV/s) and the cyclic voltammograms recorded at various scan rates are shown in Fig. 5a. The peak separation was found to be 60 mV indicating Nernstian reversibility and the relationship between peak height and the square root of scan rate found to be linear confirming conventional diffusion limited behaviour. The voltammetric profile contrasts the behaviour typically observed with solid carbon and screen printed carbon where secondary modifications such as plasma treatment [25, 26] or electrochemical anodisation [19, 22] are normally required to improve performance through exfoliation of the graphite lattice and the incorporation of greater oxygen functionality at the interface [22]. This is supported when examining the Raman spectrum of the LIG substrate (Fig. 5b) which exhibited the characteristic D and G peaks at 1350 and 1585 cm^−1^ respectively [27–29]. An intense D peak was observed which is an indicator of a high level of defects which is corroborated by the SEM images in Fig. 3. The presence of the sharp 2D peak at 2698 cm^−1^ also confirmed the formation of a small number of graphene layers [29]. The D/G ratio of 0.89 + / − 0.04 (*N* = 5) is consistent with an increased number of edge planes and is similar to literature reports of LIG [27–29] and electrochemically exfoliated carbon fibre [22]. Laser processing appears to achieve this modification at the point at which the carbon is generated and thereby provides a procedurally simple and scalable means of generating highly active electrode surfaces. It can be seen that there is a marked increase in the intensity of the peaks upon completing a double scan fabrication process and could be attributed to the increased etch depth observed in Fig. 4 creating a higher proportion of the carbonised material.

### Measurement of pH

While the laser processing of the LIG yielded a well-defined reversible voltammetric profile for ferrocyanide, the unmodified substrate itself does not provide any direct indication of the pH of the solution. In order to facilitate the latter, the electrode was modified with riboflavin. There is an extensive literature base dedicated to the detection of riboflavin [30, 31] but it has also been employed as a versatile redox probe for various analytes and biomarkers [32–36]. It can be directly tethered to an electrode via entrapment [37], covalent coupling [38] or electrodeposited [39, 40] to yield a reagentless sensor. In the case of electropolymerisation, modification of the redox centre (and hence its biocompatibility) occurs which could be problematic for in vivo sensing where leaching into the sample matrix could occur. Moreover, the large overpotentials required to polymerise riboflavin can damage the redox group and hence reduce the effectiveness of the analytical signal used to monitor pH [39, 40].

**Figure 6:**
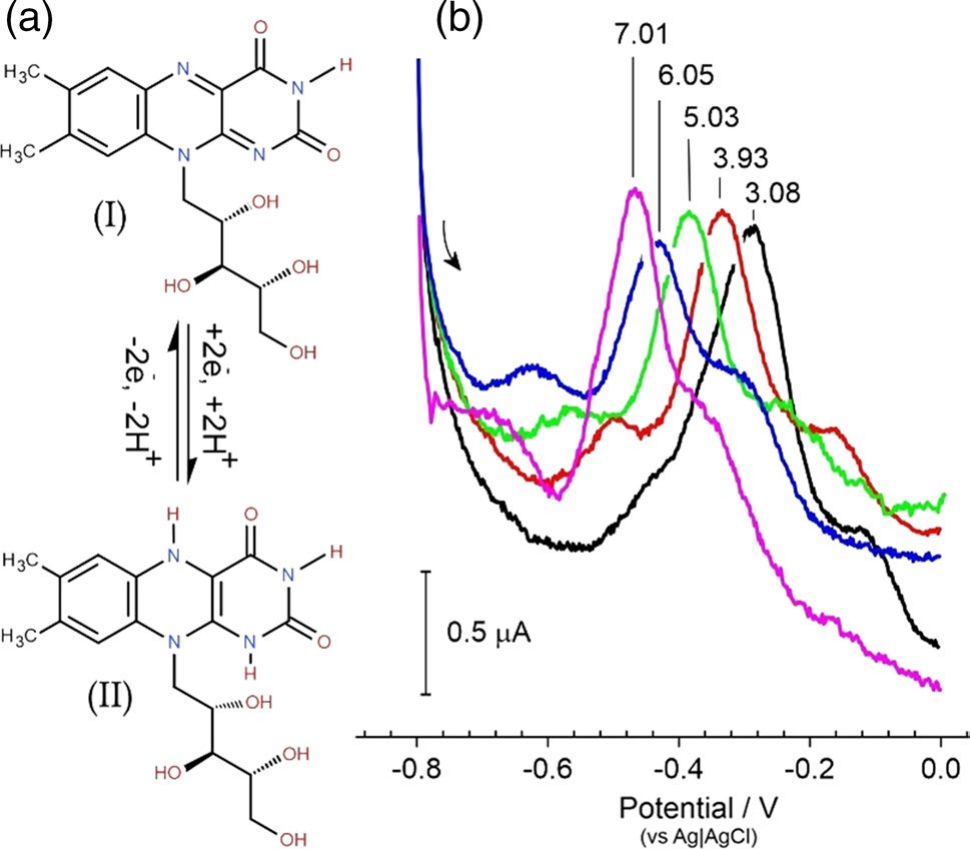
**a** Redox process associated with riboflavin. **b** Square voltammogram detailing the response of a riboflavin modified LIG electrode in Britton Robinson buffers of varying pH.

Riboflavin has however been shown to adsorb readily to carbon-based electrodes [21, 41–43] and exhibits pH dependent electrochemistry [21, 36, 42] as indicated in Fig. 6a. The peak potentials attributed to riboflavin would therefore be expected to move with changes in solution pH. Square wave voltammograms detailing the response of the LIG-Riboflavin modified electrode towards varying pH are compared in Fig. 6b. Each scan was initiated at − 0.8 V whereupon the adsorbed riboflavin is immediately reduced (Fig. 6aI → II). As the potential is swept towards less negative values, the riboflavin is re-oxidised (II → I) and gives rise to a peak profile. The position of the peak (*E*_pa_) is dependent on the solution pH and it can be seen from the voltammograms in Fig. 6b that the peak moves towards more negative potentials with increasing pH.

**Figure 7:**
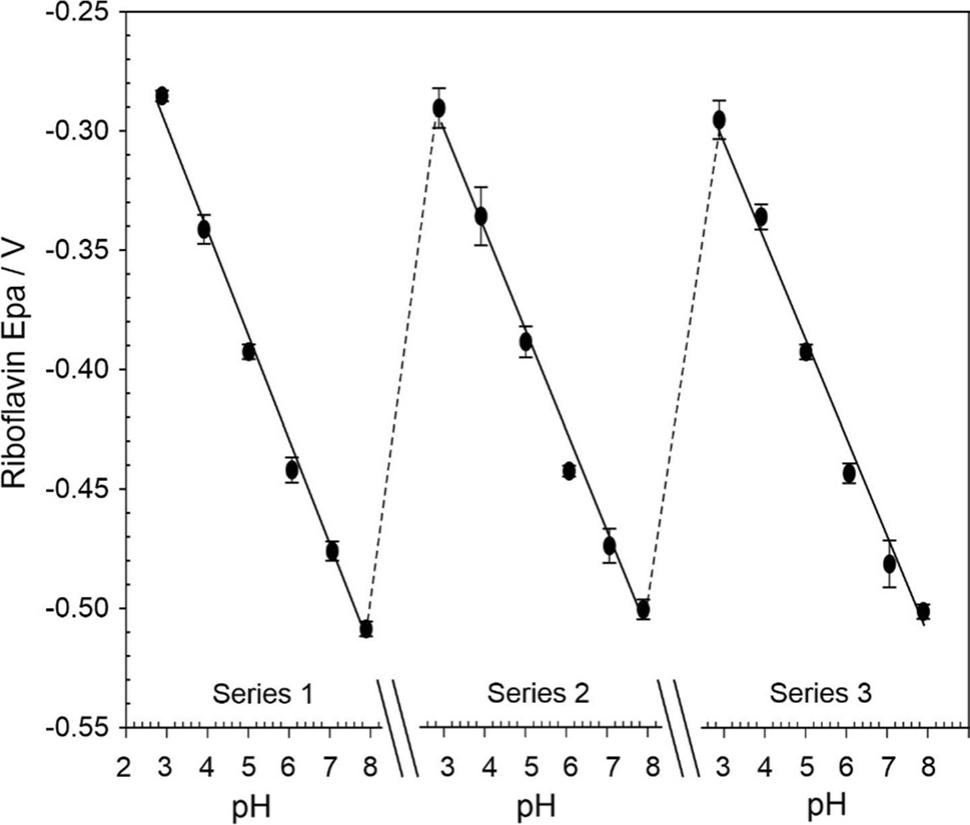
Variation of riboflavin oxidation peak (*E*_pa_) with successive exposure to Britton Robinson buffer of varying pH.

A more quantitative examination is detailed in Fig. 7 where the peak position can be seen to exhibit a linear relationship with pH with minimal drift upon commencing a second and third consecutive series of pH measurements. The simple physisorption of the riboflavin onto the LIG electrode provides pH sensitivity to the latter and benefits from being within a cathodic region of the potential window. As such, it deftly avoids the anodic signatures of ascorbate and urate whose overlapping peak processes at the LIG substrate would normally compromise the integrity of the voltammetric signal [19]. The lack of any direct covalent tethering of the riboflavin to the LIG surface, however, allows for the possibility of desorption leading to a loss of signal. This would clearly be problematic when considering the application of the drain insert sensor as a mean of periodically monitoring the pH over a number of days. The influence of repetitive scanning on the magnitude of the riboflavin peak was also assessed but in general the peak was found to be largely robust with only a 30% loss in the signal observed after 54 scans. It is unlikely that it would be necessary to continuously monitor the pH and given a recommended pouch wear time of 3–5 days [18], this would comfortably allow the device to scan every 2–3 h without any appreciable loss in the signal. It is important to note that the operation of the sensor in this instance relies on the ability to measure the peak potential and not the peak magnitude. As such, the decrease in the peak should not influence the pH analysis—until the point where it has been completely lost from the surface.

### Bacterial monitoring

**Figure 8:**
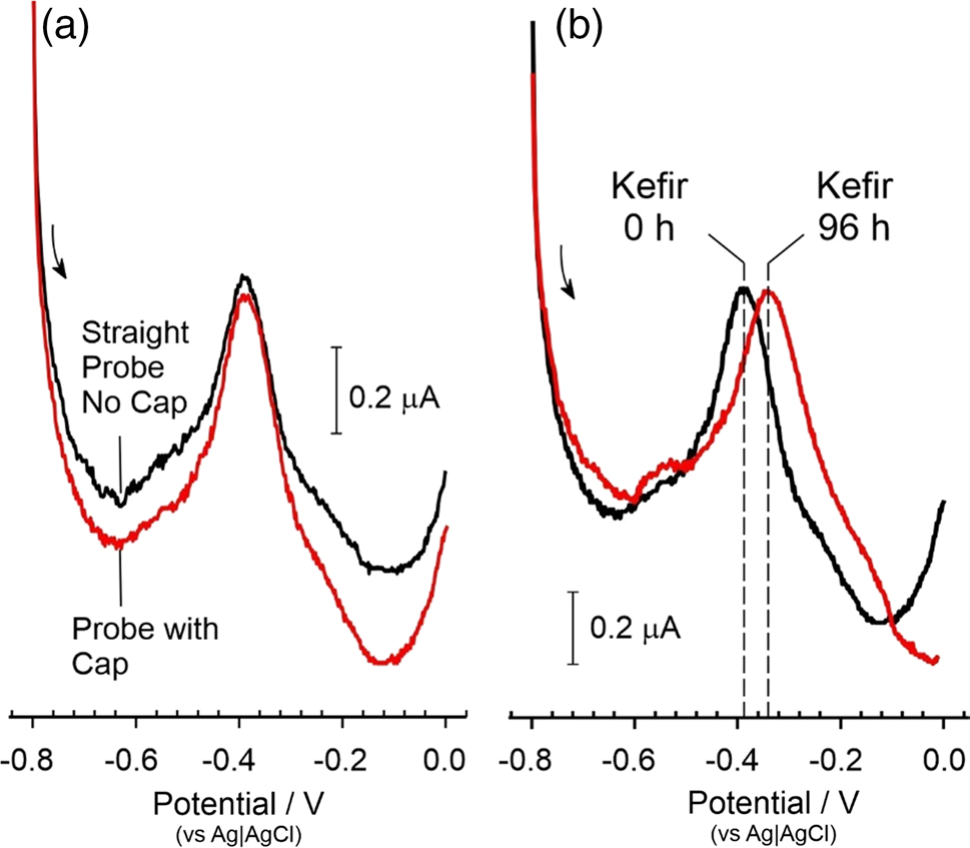
**a** Square wave voltammograms highlighting the response of the 2-electrode LIG-riboflavin probe in a heterogeneous kefir suspension before and after the addition of the protective cap and **b** comparing the response after incubation for 96 h.

The measurement of pH within well-defined buffers does not provide a realistic challenge for the sensor and, as noted, the sample matrix within which it is intended to operate is liable to be a complex mixture of nutrients, digestive by-products and bacteria. In order to facilitate a more authentic matrix, the 2-electrode riboflavin-LIG probe was placed within a fermentation broth containing kefir grains suspended within a total parenteral nutrition solution (TPN). Kefir grains are clusters of glucogalactan exopolysaccharides (kefiran) whose gel like consistency encompass a symbiotic mixture of bacteria and yeasts [44, 45]. The TPN solution contains a spectrum of macro and micronutrients of which the prime electroactive interferents ascorbate, cysteine, tryptophan and tyrosine are present at physiologically relevant concentrations (the detailed composition of the TPN solution is contained within the supplementary information: Table S1). Square wave voltammograms comparing the responses of the 2-electrode riboflavin-LIG probe in the kefir suspension with and without the addition of the protective cap are detailed in Fig. 8a. In both cases, well defined peaks (-0.391 V) were observed which is consistent with the responses observed in the Britton-Robinson buffer systems. A crucial point here is that there is little difference in the voltammetric peak profile despite that fact that the initial scan (without the cap) was done with a straight probe extending directly into the solution whereas the addition of the cap results in the electrode tip being bent through 90 degrees (as indicated in Fig. 2c). The addition of the cap was intended as protective barrier to minimise any damage to the probe occurring through physical/mechanical movements of the pouch or solids passing through the stoma. The kefir suspension was incubated at room temperature (22 °C) over 96 h during which it was anticipated that fermentation of the TPN would occur, and the pH would decrease [19, 44–46]. A second series of pH scans were then conducted after 96 h period and square wave voltammograms comparing the initial and 96 h scan detailed in Fig. 8b. While the peak profile remains largely unchanged and highlights the robustness of the actual redox probe, the position of the peak after 96 h fermentation has shifted to less negative potentials (− 0.391 to − 0.382 V) and, by inspection of the calibration data shown in Fig. 6b, would be consistent with acidification of the mixture.

The pH of the kefir solution before and after the 96 h fermentation was measured using a conventional glass pH probe and compared with the pH calculated from the peak positions observed with the 2-electrode LIG-Riboflavin probe (with integrated Ag|AgCl reference). The latter was obtained using the calibration data obtained from Fig. 7 where *E*_pa_ (/V) = − 0.0433 pH − 0.1703 (*N* = 54; *R*^2^ = 0.994) and the results compared in Table 2.

**Table 2:** Preliminary validation of pH sensing performance in a bacterial fermentation mixture

	Measured pH	Probe calculated pH	*N*
Kefir 0 h	5.3	5.02 ± 0.02	3
Kefir 96 h	3.66	3.65 ± 0.10	5

It can be seen that the measurement of pH within the simulated pouch fluid is in good agreement with the commercial probe despite the complexity of the solution. It should be noted that LIG substrates have been previously reported to minimise microbial fouling and would be advantageous in this application where a profusion of bacterial species will be commonplace. Moreover, the voltammetric profiles shown in Fig. 8 demonstrate that there is no interference from the TPN components. This would be in marked contrast to some of the voltammetric pH techniques relying on redox probes operating with the anodic region.

### Performance comparison

The key performance metrics of the system developed here are compared with recent potentiometric [48–65] and voltammetric systems [19, 66–72] in Table 3**.** The electrode response of the LIG-Riboflavin electrode is sub-Nernstian at − 43 mV/pH which is in contrast to the − 59 mV/pH observed with solution-based riboflavin [21, 36, 42], and polymer bound flavin [19] and flavanone [72] derivatives. Similar non Nernstian gradients were found with the LIG-Riboflavin system when switching between 2-electrode and 3-electrode configurations. It is noteworthy that Srinivas et al. (2022) also observed a similar non Nernstian (2H^+^/3e^−^) behaviour (45 mV) with a carbazole-quinone derivative and attributed the response to the orientation/interaction of the redox probe on the surface [70]. The change in behaviour upon being surface confined is supported by the work by Lee et al. (2013) where the pKa and hence protonation of the redox species can be modified by the surface interactions [73]. Nevertheless, it can be seen from Table 2 that while the sensitivity of the probe may be reduced, the accuracy is retained. It must also be noted that the Ag|AgCl pseudo reference will be dependent on the chloride ion which, in this scenario, will be a natural constituent of the ileostomy fluid and is typically maintained at ~ 0.1 M [74, 75].

**Table 3:** Comparison of electrochemical pH measurement systems

Modifier	Type	Sensitivity mV/pH	pH range	Test medium	References
CeTi_x_O_y_	P	90	2–12	N/S	[48]
RuO_2_/Nafion	P	55	2–6	Beverages	[49]
ERGO PANI/Nafion	P	55	2–9	Fermentation	[50]
Graphite/polyurethane	P	11	5–9	Sweat	[51]
WO_4_/WO_3_	P	56	2–10	N/S	[52]
NiO	P	63	1–13	N/S	[53]
ZnO/W	P	46	2–9	CSF	[54]
Ni_3_(PO_4_)_2_·8H_2_O	P	35	4–7	Sweat	[55]
Pt-IrO_x_	P	56	4–9	Biofilm	[56]
ZnO	P	43	2–9	Tumour Cells	[57]
PANI/3D Printed MN	P	59	4–9	ISF	[58]
LIG/PANI/PtNp	p	72	4–8	Sweat	[59]
IrO_x_/Si–Si_3_N_4_/PDMS	P	83	2–12	Tap Water	[60]
Au IDE/PANI	P	69	4–9	Sweat	[61]
TiN/Nafion	P	58	2–12	N/S	[62]
PANI/Graphite	P	53	3–10	N/S	[63]
TiO_2_–SnO_2_	P	64	2–12	Beverages	[64]
Sb/Sb_2_O_3_	P	42	4–9	Lake Water	[65]
Carbon-quinone	V	73	2–8	Saliva	[66]
PANI	V	50	4–10	Wound fluid	[67]
Poly Dopamine	V	58	1–12	N/S	[68]
Di-Feruloyl sesamol/CB SPE	V	59	3–11	Urine/Saliva	[69]
SPE/MWCNT/Car-HQ	V	48	2–11	Urine/Saliva	[70]
Chitosan–butein/Carbon Gauze	V	25	5–9	N/S	[71]
2′-Hydroxyflavanone/Nafion	V	55	4–9	Seawater	[72]
Poly Flavin	V	55	2–8	Kefir	[19]
LIG Riboflavin (Solution)	P/V	24/56	2–8	SWF	[21]
LIG-Riboflavin	V	43	2–8	Kefir	This Work

*P* potentiometric, *V* voltammetric, *ERGO* Electrochemically reduced graphene oxide, *PANI* polyaniline, *SPE* screen printed electrode, *MN* microneedle, *IDE* interdigitated electrode, *CB* carbon black, *LIG* laser induced graphene, *MWCNT* multiwalled carbon nanotube, *Car-HQ* Benzyl-3-bromo-1H-carbazole-1,4(9H)-dione, *PtNp* platinum nanoparticles, *ISF* Interstitial fluid; *CSF* cerebral spinal fluid, *SWF* simulated wound fluid

A brief inspection of the sensing configurations/modifier employed Table 3 highlight considerable complexity and stand in contrast to the relative simplicity and inherent biocompatibility of the LIG-Riboflavin system proposed here.

## Conclusions

Given the complexity of the surgical interventions inherent in ostomy creation and the complex microbiochemical—physiological interactions that arise in daily management, there is a pressing need for smarter systems that can provide point of care diagnostic information. As yet, there are few sensing options but the arrival of lasered graphene substrates could open new avenues for exploration. In exploiting the drain port, the sensor can be incorporated into the pouch without disrupting the normal adhesive function of the baseplate which secures the pouch to the abdomen. As such, the device does not compromise the performance of the pouch. The LIG-based probes highlighted here demonstrate the robustness of such devices for measuring pH, but the underpinning sensor could easily be adapted to take advantage of the many assays that have been previously developed for carbon-based electrodes.

## Supporting information

The detailed technical specifications and STL file of the 3D printed sensor are available along with electron microscopic analysis of the print quality. The composition of the total parenteral nutrition solution used during the incubation of the Kefir mixture is also specified.

### Supplementary Information

Below is the link to the electronic supplementary material.

Supplementary file1 (DOCX 1702 KB)
