# Supplementary material for: Moving towards in pouch diagnostics for ostomy patients: exploiting the versatility of laser induced graphene sensors
Source: J Mater Sci. 2023 Sep 8;58(35):14207–19. doi: 10.1007/s10853-023-08881-x (PMC10511578; doi:10.1007/s10853-023-08881-x)
Supplement: Supplementary file 1 — Supplementary file1 (DOCX 1702 KB) [file 10853_2023_8881_MOESM1_ESM.docx]

**Laser induced graphene composites for monitoring ileostomy fluid pH: Towards in-pouch diagnostics**

Conor McCann^1^, Victoria Gilpin^1^, Cameron Scott^1^, L.Kirsty Pourshahidi^2^, Chris. Gill^2^ and James Davis^[[1]](#footnote-1)^*

^1^School of Engineering, Ulster University, Belfast, Northern Ireland

^2^School of Biomedical Sciences, Ulster University, Coleraine, Northern Ireland

**Supporting Information**


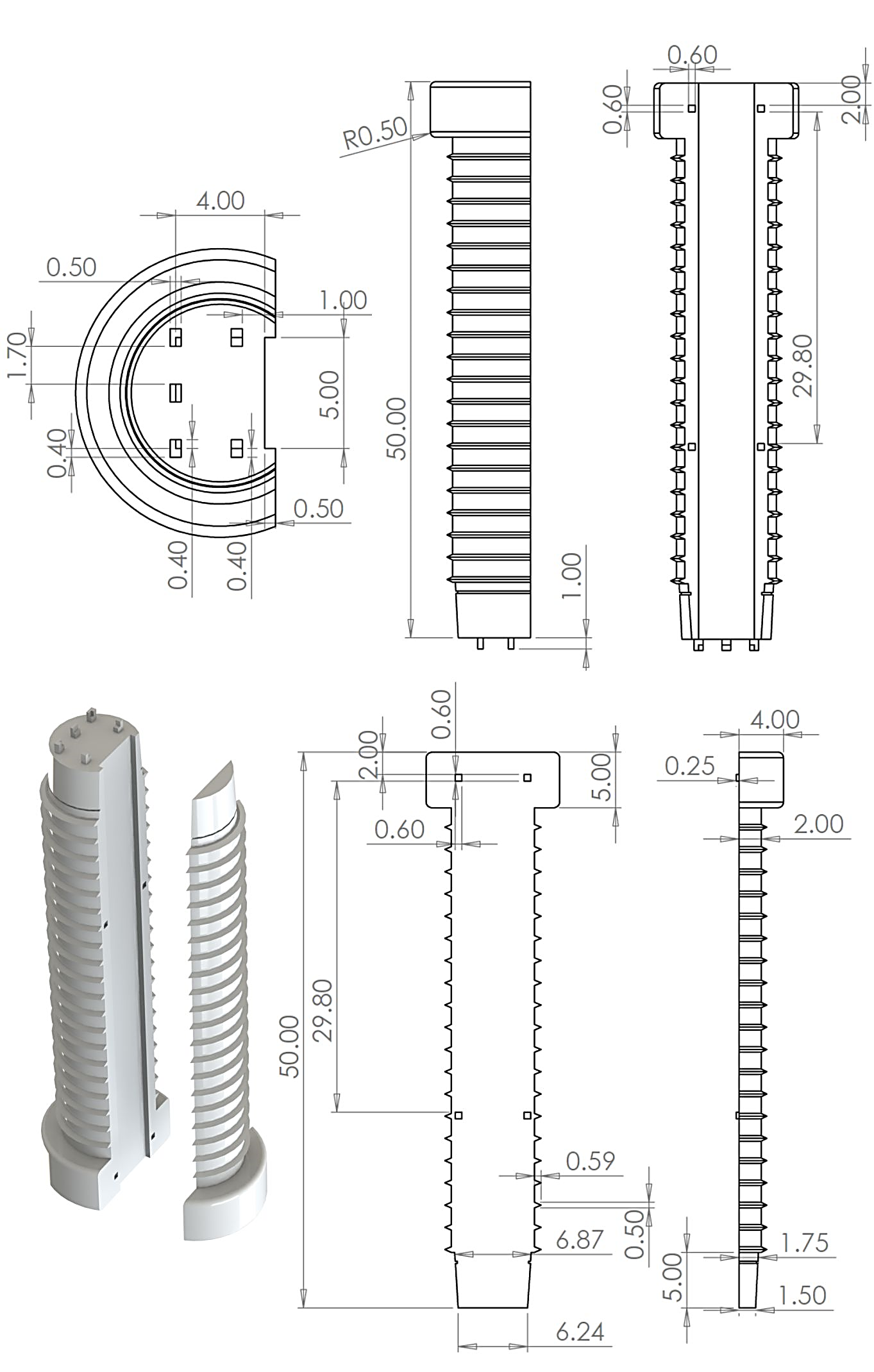


**Figure S1.** Technical specifications of the pouch probe


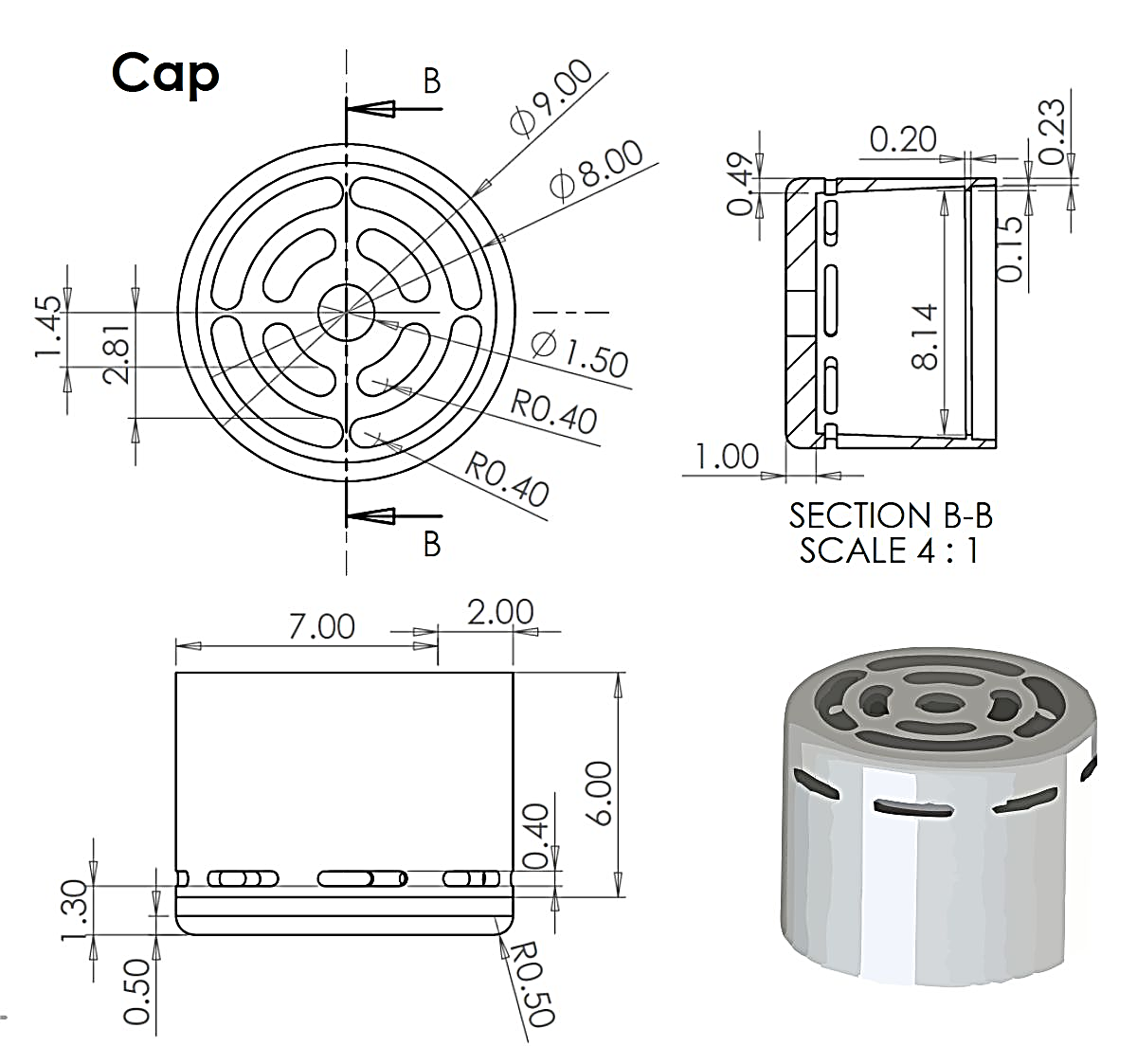


**Figure S2.** Technical specifications of the probe cap


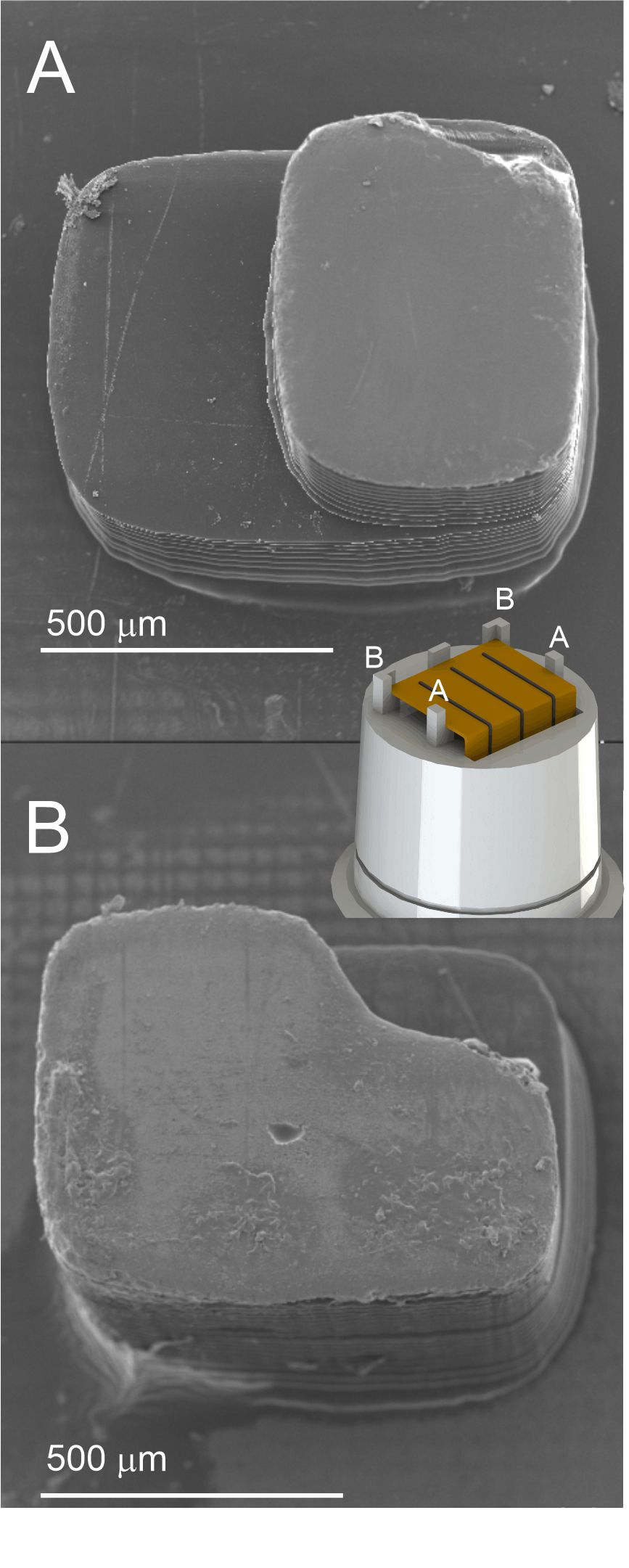


**Figure S3.** Scanning electron micrographs of the UV-LCD resin printed pillars designedto support the LIG substrate.

**Table S1.** Composition of the total parenteral nutrition (TPN) solution used for the fermentation of Kefir.

1. To whom correspondence should be addressed. T: +44(0)28 903 66407; E: james.davis@ulster.ac.uk [↑](#footnote-ref-1)
